# Interferon-γ-induced upregulation of immunoproteasome subunit assembly overcomes bortezomib resistance in human hematological cell lines

**DOI:** 10.1186/1756-8722-7-7

**Published:** 2014-01-13

**Authors:** Denise Niewerth, Gertjan JL Kaspers, Yehuda G Assaraf, Johan van Meerloo, Christopher J Kirk, Janet Anderl, Jonathan L Blank, Peter M van de Ven, Sonja Zweegman, Gerrit Jansen, Jacqueline Cloos

**Affiliations:** 1 Department of Pediatric Oncology/Hematology, VU University Medical Center, Amsterdam, The Netherlands; 2 The Fred Wyszkowski Cancer Research Lab, Technion-Israel Institute of Technology, Haifa, Israel; 3 Research Department, Onyx Pharmaceuticals, South San Francisco, CA, USA; 4 Discovery, Millennium Pharmaceuticals Inc., Cambridge, MA, USA; 5 Department of Hematology, VU University Medical Center, Amsterdam, The Netherlands; 6 Department of Epidemiology and Biostatistics, VU University Medical Center, Amsterdam, The Netherlands; 7 Department of Rheumatology, VU University Medical Center, Amsterdam, The Netherlands

**Keywords:** Leukemia, Proteasome, Immunoproteasome, Proteasome inhibitor, Bortezomib, Interferon-gamma

## Abstract

**Background:**

Despite encouraging results with the proteasome inhibitor bortezomib in the treatment of hematologic malignancies, emergence of resistance can limit its efficacy, hence calling for novel strategies to overcome bortezomib-resistance. We previously showed that bortezomib-resistant human leukemia cell lines expressed significantly lower levels of immunoproteasome at the expense of constitutive proteasomes, which harbored point mutations in exon 2 of the *PSMB5* gene encoding the β5 subunit. Here we investigated whether up-regulation of immunoproteasomes by exposure to interferon-γ restores sensitivity to bortezomib in myeloma and leukemia cell lines with acquired resistance to bortezomib.

**Methods:**

RPMI-8226 myeloma, THP1 monocytic/macrophage and CCRF-CEM (T) parental cells and sub lines with acquired resistance to bortezomib were exposed to Interferon-γ for 24-48 h where after the effects on proteasome subunit expression and activity were measured, next to sensitivity measurements to proteasome inhibitors bortezomib, carfilzomib, and the immunoproteasome selective inhibitor ONX 0914. At last, siRNA knockdown experiments of β5i and β1i were performed to identify the contribution of these subunits to sensitivity to proteasome inhibition. Statistical significance of the differences were determined using the Mann-Whitney U test.

**Results:**

Interferon-γ exposure markedly increased immunoproteasome subunit mRNA to a significantly higher level in bortezomib-resistant cells (up to 30-fold, 10-fold, and 6-fold, in β1i, β5i, and β2i, respectively) than in parental cells. These increases were paralleled by elevated immunoproteasome protein levels and catalytic activity, as well as HLA class-I. Moreover, interferon-γ exposure reinforced sensitization of bortezomib-resistant tumor cells to bortezomib and carfilzomib, but most prominently to ONX 0914, as confirmed by cell growth inhibition studies, proteasome inhibitor-induced apoptosis, activation of PARP cleavage and accumulation of polyubiquitinated proteins. This sensitization was abrogated by siRNA silencing of β5i but not by β1i silencing, prior to pulse exposure to interferon-γ.

**Conclusion:**

Downregulation of β5i subunit expression is a major determinant in acquisition of bortezomib-resistance and enhancement of its proteasomal assembly after induction by interferon-γ facilitates restoration of sensitivity in bortezomib-resistant leukemia cells towards bortezomib and next generation (immuno) proteasome inhibitors.

## Background

Bortezomib is a tight binding yet reversible proteasome inhibitor that is indicated for treatment of newly diagnosed and relapsed multiple myeloma (MM) [1], and is currently being tested in clinical trials for childhood leukemia [2]. In July 2012, the epoxyketone-based proteasome inhibitor carfilzomib [3] was approved in the US for patients with relapsed and refractory MM who received at least two prior therapies (including bortezomib and an immunomodulatory agent) and progressed on or within 60 days of completion of the last therapy [4,5]. Notwithstanding promising initial results, acquired resistance to bortezomib is an emerging factor, which may limit its efficacy in the treatment of hematologic malignancies. The clinical impact of acquired resistance has been demonstrated in poor responses of MM patients who were re-treated with bortezomib [6]. Although bortezomib-retreatment was effective, the response rates as well as the duration of response were decreased as compared to initial therapy, which may point to the development of bortezomib-resistance in (a subgroup of) patients [7]. To investigate possible mechanisms of bortezomib resistance, we previously developed *in vitro* cell line models of hematologic malignancies in which acquired resistance to bortezomib was provoked by chronic exposure to gradually increasing bortezomib concentrations [8,9]. These bortezomib-resistant cell lines were characterized by an increased expression of the constitutive proteasome subunit β5 harboring mutations in the bortezomib-binding pocket, along with a decreased expression of non-mutated immunoproteasome subunits. Furthermore, these bortezomib-resistant cells displayed cross-resistance to other proteasome inhibitors that target β-subunits of the proteasome [9]. The constitutive proteasome has three proteolytically active subunits; β5 (*PSMB5*), β1 (*PSMB6*), and β2 (*PSMB7*) which harbor the chymotrypsin-like, caspase-like, and trypsin-like catalytic activities, respectively. Upon exposure to inflammatory cytokines, including interferon-γ (IFN-γ) or tumor necrosis factor α (TNF-α), the constitutive subunits are exchanged for immunoproteasome subunits β5i (LMP7) β1i (LMP2), and β2i (MECL-1) [10]. While β5i harbors chymotrypsin-like activity as in β5, whereas β2i and β2 contain trypsin-like activity, β1i displays chymotrypsin-like activity rather than β1-associated caspase-like activity [11,12]. The immunoproteasome is dominantly expressed in cells of hematologic origin and its primary function was originally attributed to improve MHC Class I antigen presentation. To this end, the immunoproteasome can produce a distinct set of peptides from the constitutive proteasome because the immunoproteasome cleaves preferably after hydrophobic and basic amino acids (cleaved by chymotrypsin-like and trypsin-like activities, respectively) that can better fit MHC Class I molecules [13]. Therefore, peptides generated by the immunoproteasome may be more efficient in T-cell activation than peptides from the constitutive proteasome. In addition, Seifert and colleagues [14] provided evidence to implicate the immunoproteasome in protein degradation after immune response-induces stress, and that the immunoproteasome is more efficient than the constitutive proteasome in controlling the protein degradation process. However, this property of immunoproteasomes was recently challenged and warrants further investigations [15,16]. Several studies [17-19] have reported higher immunoproteasome expression compared to constitutive subunits in B-cell malignancies, underscoring the potential importance of the immunoproteasome in the homeostasis of hematologic diseases [20]. However, although there is evidence for pre-clinical activity of the β5i-specific proteasome inhibitor ONX 0914 [21] in experimental autoimmune disease models, data justifying its use for the treatment of hematologic malignancies is limited [2].

Tumor cells have the capacity to modulate immunoproteasome function to escape immune surveillance [22]. This condition may also arise in hematologic tumor cells with acquired resistance to bortezomib due to the acquisition of mutations in the *PSMB5* gene encoding the constitutive β5 subunit. Since its immunoproteasome β5i counterpart does not harbor mutations, downregulation of immunoproteasome in bortezomib-resistant hematologic tumor cell lines may provide a mechanism to escape targeting by bortezomib. From a therapeutic perspective, this would imply that tipping the balance towards upregulation of immunoproteasome expression could re-confer sensitivity to bortezomib or next generation proteasome inhibitors designed to target immunoproteasomes [20,23].

Original studies by Altun et al [24] showed that inflammatory cytokines such as IFN-γ and TNFα were efficient inducers of immunoproteasomes in MM cell lines, including 8226 cells. Functional studies by Busse et al [25] showed that exposure to IFN-γ enhanced bortezomib-sensitivity in B-cell lines by 50%, for which the underlying mechanism was unexplored. Furthermore, the β5i immunoproteasome subunit played a critical role in IFN-γ-induced apoptosis by degradation of Mcl-1 in atherosclerotic lesion-derived cells [26].

In this study, we explored whether IFN-γ-induced upregulation of immunoproteasome expression in bortezomib-resistant leukemia cell lines in which both immunoproteasome expression is suppressed and mutated β5 subunits are overexpressed can serve as a therapeutic strategy to restore sensitivity towards bortezomib, carfilzomib and ONX 0914.

## Methods

### Cell culture

Human T-cell ALL CCRF-CEM cells, human myeloid leukemia THP1 cells, and human multiple myeloma RPMI-8226 cells (ATCC, Manassas, VA, USA) were cultured in RPMI-1640 medium containing 2 mM glutamine (Invitrogen/Gibco, Carlsbad, CA, USA) supplemented with 10% fetal calf serum (Greiner Bio-One, Alphen a/d Rijn, The Netherlands) and 100 μg/ml penicillin/streptomycin (Invitrogen) at 5% CO_2_ and 37°C. Cell cultures were seeded at a density of 3×10^5^ cells/ml and refreshed twice weekly. Bortezomib-resistant sublines of these cell lines were established previously [8,9]. Authenticity of bortezomib-resistant and parental cell lines was verified by STR marker analysis for D12S1045, D8S1132, D19S253, and D17S1293.

### Antibodies, drugs and reagents

Antibodies to proteasome subunits β1, β2, β5, β1i, and β5i were purchased from Enzo Life Sciences (Farmingdale, NY, USA). In addition, anti-actin (clone C4) was purchased from Millipore (Temecula, CA, USA), anti-NOXA antibody from Abcam (Cambridge, UK), anti-ubiquitin (P4D1) from Santa Cruz Biotechnology (Santa Cruz, CA, USA) and the IRDye infrared secondary labeled antibodies was from LI-COR Biosciences (Lincoln, NE, USA). Bortezomib was provided by Millennium Pharmaceuticals (Cambridge, MA, USA). The epoxyketone-based proteasome inhibitors carfilzomib and ONX 0914 were provided by Onyx Pharmaceuticals, Inc. (South San Francisco, CA, USA). IFN-γ was purchased from Sanquin (Amsterdam, the Netherlands).

### Proteasome active site ELISA

An ELISA-based assay (Pro-CISE) for quantitative assessment of active constitutive and immunoproteasome subunits was performed as previously described [27]. Briefly, cell lysate was incubated with a biotinylated proteasome active-site binding probe. Lysate was then denatured, and subunits bound to probe were isolated with streptavidin-conjugated sepharose beads. Individual subunits were probed with subunit-specific primary antibodies, followed by HRP-conjugated secondary antibodies. A chemiluminescent substrate was used to generate signal associated with HRP binding, which was read on a luminometer. Absolute values of nanograms of subunit per microgram of total protein were based on a purified proteasome standard curve. Protein quantification was performed utilizing the Pierce BCA Protein Assay (Thermo Scientific, Rockford, IL, USA).

### Lightcycler quantitative PCR

Lightcycler® 480 SYBR Green I Master was used to quantify expression levels of mutated and unmutated allele in the bortezomib-resistant cell lines. Primers specific for the Ala49Thr mutation, primers specific for the unmutated allele, and primers for total *PSMB5* were developed (Tib-molbiol, Germany) (Additional file 1). GUS was used as housekeeping gene. All primers were used at 0.5 μM each. 5 μl of cDNA template was added to the PCR mix. Results were analysed by advanced relative quantification using the comparative cycle time (Ct) method by Lightcycler 480 Instrument Software version 1.5 (Roche Diagnostics, Switzerland).

### Cell growth inhibition assay

*In vitro* drug sensitivity was determined using the 4-day MTT cytotoxicity assay [28]. Prior to these experiments, bortezomib-resistant cells were cultured in bortezomib-free medium for at least 4 days. Cells were then pre-exposed for 48 h to 100 U/ml IFN-γ and then subjected to various concentrations of bortezomib (range: 0.001 μM – 2 μM), CFZ (0.008 nM - 15.6 nM for the parental cell lines; 0.0005 μM - 1 μM for bortezomib-resistant lines), or ONX 0914 (0.008 μM – 16 μM) for 4 days. For siRNA experiments, cells were incubated with 100 nM siRNA for 24 h before adding 100 U/ml IFN-γ for 48 hours, followed by the same concentration ranges of the drugs as specified above. The IC_50_ value was defined as the drug concentration necessary to inhibit 50% of the cell growth compared to growth of the untreated control cells.

### Proteasome activity

#### Intact-cell based caspase-like, trypsin-like, and chymotrypsin-like proteasome activities

An intact cell-based assay to measure basal and IFN-γ-induced upregulation of caspase-like, trypsin-like, and chymotrypsin-like proteasome activities was conducted by using a Proteasome-Glo assay kit according to the manufacturer’s instructions (Promega, Madison, WI). Before determination of proteasome activity, cells were exposed to 100 U/ml IFN-γ for 24 h, 48 h, 72 h, and 96 h at 37°C in a white flat-bottomed 96-well plate (Greiner Bio-one, The Netherlands) at a density of 10 000 cells per well in a total volume of 50 μl. After 15-min incubation period with luminogenic substrates, luminescence was determined with an Infinite 200 Pro microplate reader (Tecan, Giessen, The Netherlands). Background measurements of cell-free medium plus substrate were subtracted from cell measurements.

#### HLA Class I expression

HLA Class I expression was determined using HLA-ABC FITC antibody (5 μg/ml) (eBioscience, San Diego, CA, USA) and mouse IgG2a antibody (5 μg/ml) as isotype control. Cells were measured on the FACSDiva, and analyzed using CELLQUEST software.

#### Specific β5, β5i, and β1i subunit activities in cell extracts

For measurement of specific β5, β5i, and β1i activities, the Ac-WLA-AMC, Ac-ANW-AMC, and Ac-PAL-AMC fluorogenic substrates were used, respectively [29]. Cells were washed in ice-cold phosphate-buffered saline and 5 mM ethylenediaminetetraacetic acid (EDTA) was added at pH 8.0 and samples were frozen at -80°C until analysis. Samples were thawed and centrifuged at 10,000 g for 10 minutes at 4°C. The supernatant was removed and assayed for protein content using the BioRad Protein Assay following the manufacturer's protocol (BioRad, Hercules, CA, USA).

Assays were performed at 37°C in a final volume of 200 μL using 96-well black opaque plates (Greiner bio-one, Germany). Protein extracts were diluted to 200 μg/mL in 5 mM EDTA at pH 8.0. Diluted protein extract aliquots (50 μL) were dispensed per well, giving 10 μg of protein extract per reaction. Reactions were initiated by addition of 150 μL of 133 μM peptide-AMC substrate in 20 mM N-[2-Hydroxyethyl]piperazine-N-[2-ethanesulfonic acid] (HEPES), pH 7.4, containing 0.5 mM EDTA. Peptidase activity was measured by kinetic monitoring of 7-amino-4-methylcoumarin (AMC) production over two hours with a Biotek plate reader (Winooski, VT, USA) and analyzed by GraphPad Prism software (La Jolla, CA, USA) with linear regression analysis.

### RNA interference

For RNA interference experiments all targeted and non-targeted siRNA constructs were obtained from Dharmacon (Lafayette, USA) and all experiments were performed in 6well plates. THP1/BTZ200 cells were cultured following the DharmaFECT general transfection protocol conditions for THP1 cells. Briefly, prior to transfection, cells were cultured overnight at a density of 3 × 10^5^ cells/ml in RPMI-1640 medium supplemented with 10% FCS. Cells were transfected using Dharmafect 2 and 100 nM of *PSMB8* or *PSMB9* ON-TARGETplus SmartPool siRNA. As negative control 100 nM ON-TARGETplus siControl non-targeting siRNA was used and the GAPDH siRNA pool was included as a positive control. The transfection methods had no effect on cell growth. At several time points, transfection-efficiency was determined by mRNA expression analysis. 24 h after siRNA transfection when >80% knockdown was established, IFN-γ (100 U/ml) was added for 24 h (or 48 h when indicated), followed by bortezomib administration.

### Protein expression/Western blotting

Protein expression of proteasome subunits was determined by Western blotting, as previously described [9]. Protein bands were quantified by Odyssey software, corrected for background, and normalized by β-actin to correct for loading differences within blots.

### cDNA synthesis of proteasome subunits and quantitative RT-PCR

After RNA isolation by utilizing the RNAeasy Mini kit (Qiagen, Valencia, CA, USA), cDNA was synthesized using RT buffer (Invitrogen), containing 5 mM DTT (Invitrogen), 2 mM dNTP (Roche), 96 μg/ml pdN6 (Roche), 0.75 U/μl M-MLV (Invitrogen) and 2 U/μl RNAsin (HT Biotechnology Ltd., Cambridge, UK). mRNA expression levels of proteasome subunits *PSMB*5, *PSMB6*, *PSMB7*, *PSMB8, PSMB9, PSMB10*, and β-glucuronidase as a reference were quantified using real-time PCR analysis (Taqman) on an ABI Prism 7700 sequence detection system (PE Applied Biosystems, Nieuwerkerk a/d IJssel, The Netherlands). For *PSMB5*, a Taqman gene expression assay was used according to the manufacturers’ instructions (Hs00605652_m1, Applied Biosystems). All other primers and probes were designed using Primer Express software (Applied Biosystems) and are indicated in Additional file 1. Probes were labeled with 5’-FAM and 3’-BHQ1 as a reporter. Real-time PCR was performed in a total reaction volume of 25 μl containing TaqMan buffer A (Applied Biosystems), 4 mM MgCl_2_, 0.25 mM of each dNTP (Amersham Pharmacia Biotech) and 1.25 U AmpliTaq Gold DNA polymerase (Applied Biosystems). Samples were heated for 10 min at 95°C to activate the AmpliTaq Gold DNA polymerase and amplified during 40 cycles of 15 s at 95°C and 60 s at 60°C. Relative mRNA expression levels of the target genes in each sample were calculated using the comparative cycle time (Ct) method. The Ct of the target gene was normalized to the GUS Ct value by subtracting the GUS Ct value from the target Ct value. The mRNA expression level for each target PCR relative to GUS was calculated using the following equation: mRNA expression = 2^(Ct target-Ct GUS)^ × 100%.

### Statistical analysis

A logistic (or Emax) model was chosen to model the sigmoid-shaped relationship between concentration of the drug and percentage growth allowing for different parameters values for the experiments without and with IFN-γ. The expected percentage growth was assumed to be of the following parametric form

$$E\left[\mathrm{\%Growth}\right]=\frac{\mathrm{Ma}x+1_{\mathrm{INF}}\mathrm{\Delta Ma}x}{1+{\left(\frac{\mathrm{concentration}}{\mathrm{IC}50+1_{\mathrm{INF}}\mathrm{\Delta IC}50}\right)}^{\mathrm{Hill}50+1_{\mathrm{INF}}\mathrm{\Delta Hill}}},$$

where 1_*INF*_ = 0 and 1 for experiments without and with IFN-γ, respectively. The parameters *Max*, *IC*_*50*_, and *Hill* model the maximum of the percentage growth, the concentration at which the percentage growth is 50% of the maximum and the slope of the curve for the experiments without IFN-γ. Parameters Δ*Max*, Δ*IC*_*50*_, and Δ*Hill* model the change in these parameters as a result of adding IFN-γ. Our models included additive random effects for replicate and a residual error which were assumed to be normally distributed and independent. Nonlinear mixed models were fitted in SAS version 9.2 separately for each drug and cell line combination.

Statistical significance of the differences in subunit expression and proteasome activity were determined using the Mann-Whitney U test. Statistical significance was achieved when P < 0.05. Statistical analyses were performed using SPSS (version 20.0).

## Results

### Characterization of bortezomib-sensitive and bortezomib-resistant hematologic tumor cell lines

**Figure 1:**
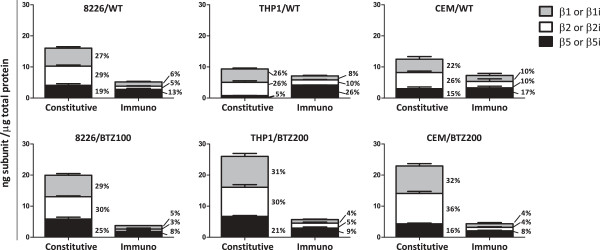
**Proteasome subunit expression in bortezomib-resistant and bortezomib-sensitive 8226 (MM), THP1 (AML) and CEM (ALL) cells.** Protein expression levels of constitutive proteasome subunits (β5, β1, and β2) and immunoproteasome subunits (β5i, β1i, and β2i) were determined by ProCISE and expressed in ng/μg total protein for 8226/WT, CEM/WT, THP1/WT, 8226/BTZ100, THP1/BTZ200 and CEM/BTZ200 cells. Percentages of individual subunits are given. Results depicted represent the mean (± SD) of 3 separate experiments.

Cell lines were of multiple myeloma (8226), T-cell leukemia (CCRF-CEM) and myelomonocytic leukemia (THP1) origin and their bortezomib-resistant sublines displayed 40-150 fold bortezomib resistance upon cell growth inhibition [8,9]. When studying the absolute protein expression in ng subunit/μg total protein of the catalytically active subunits in wild type (WT) and bortezomib-resistant sublines, all WT tumor cell lines harbored a lower amount of immunoproteasomes (β1i + β2i + β5i) than constitutive proteasomes (β1 + β2 + β5) (8226/WT: 24% *vs.* 76%, THP1/WT: 43% *vs.* 57%, and CEM/WT: 37% *vs.* 63%, respectively). All three bortezomib-resistant sublines displayed a decrease in immunoproteasome expression while an increase in constitutive subunits was observed (8226/BTZ100: 16% *vs.* 84%, THP1/BTZ200: 18% *vs.* 82%, and CEM/BTZ200: 16% *vs.* 84%, respectively) (Figure 1). Additionally, total proteasome content per μg total protein was increased in CEM/BTZ200 and THP1/BTZ200 cells compared to their WT counterparts (19.8 ng/μg *vs.* 27.3 ng/μg for CEM/BTZ200 and 16.5 ng/μg *vs.* 31.7 ng/μg for THP1/BTZ200 cells), whereas the proteasome content in 8226/BTZ100 was similar to that of 8226/WT (21.2 ng/μg *vs.* 23.7 ng/μg) (Figure 1).

### IFN-y exposure tips balances from constitutive proteasomes to immunoproteasomes

**Figure 2:**
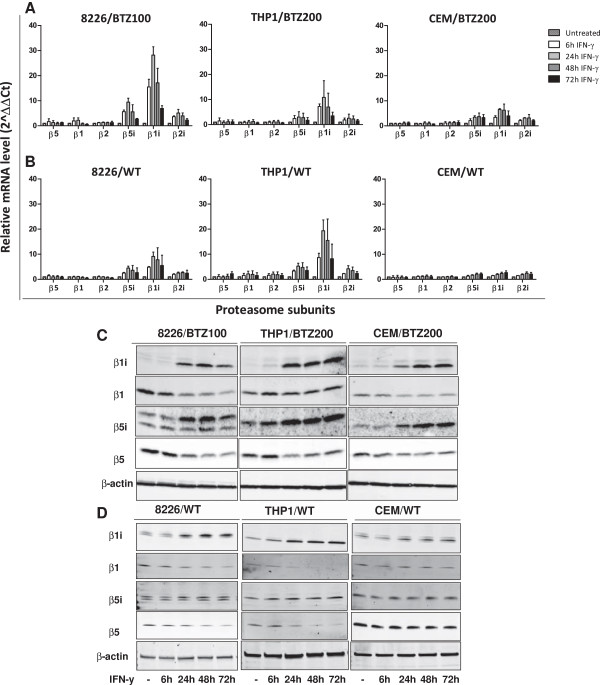
**Effect of IFN-γ exposure on constitutive and immunoproteasome subunits mRNA and protein levels in bortezomib-resistant and bortezomib-sensitive 8226 (MM), THP1 (AML) and CEM (ALL) cells. (A)** Expression levels of β5, β1, β2, β5i, β1i and β2i mRNA were monitored after 6-72 hrs exposure of 8226/BTZ, THP1/BTZ, CEM/BTZ and **(B)** their parental counter parts to 100 U/ml IFN-γ. Results are presented relative to untreated controls conditions and depicted as mean (± SD) of 3 individual experiments for bortezomib-resistant cells and mean of two experiments performed in duplicate for parental cells. **(C)** Western blot analysis of β1i, β1, β5i and β5 protein expression after 6-72 hrs exposure of 8226/BTZ, THP1/BTZ, CEM/BTZ and **(D)** their parental counter parts to 100 U/ml IFN-γ. One representative example of 3 experiments is shown.

Since low levels of immunoproteasome expression appeared to be a characteristic feature of bortezomib-resistant tumor cell lines, we aimed at increasing immunoproteasome levels by exposing cell lines to IFN-γ (100 U/ml) for 6-72 h. Indeed, IFN-γ transiently increased (peaks between 24-48 hours) mRNA levels of β5i, β1i, and β2i up to 8-fold, 30-fold and 4-fold, respectively. In contrast, constitutive subunits slightly decreased after 24 h (Figure 2A). For comparison, parental cells essentially showed a similar pattern of immunoproteasome mRNA induction, except that the degree of induction was markedly lower than in bortezomib-resistant cells; only THP1/WT cells displayed a higher induction compared to THP1/BTZ200 (Figure 2B). These findings were corroborated at the protein level using Western blot analysis (Figure 2C-D) and the ELISA-based ProCISE assay (Additional file 2), illustrating an increase in β5i β1i, and β2i subunit expression along with a decreased expression of constitutive subunits after IFN-γ exposure. To determine whether or not the mutated and/or wild type allele of *PSMB5* was downregulated, mutation-specific primers (for Ala49Thr), wild type-specific primers, and primers for total exon 2 of *PSMB5* were developed to quantify the contribution of the mutated allele in 8226/BTZ100 and THP1/BTZ200 before and after 48 hours of IFN-γ exposure. First, amplification curves showed that 8226/BTZ100 cells harbored about 3-fold lower expression of unmutated *PSMB5* mRNA compared to parental 8226/WT cells. However, the expression of total exon 2 of *PSMB5* was about 3-fold higher in 8226/BTZ100 cells compared to 8226/WT cells, which implies that mutated Ala49Thr in the resistant cells outweighs unmutated *PSMB5* (Additional file 3). Similar results were observed for THP1 cells, although THP1/BTZ200 cells expressed slightly higher levels of unmutated *PSMB5* compared to THP1/WT (Additional file 3). Furthermore, expression of these *PSMB5* variants was determined in the resistant cell lines exposed to IFN-γ. Of note, total *PSMB5* expression was decreased in cells exposed to IFN-γ compared to unexposed cells (8226/BTZ100; 24% decrease, and for THP1/BTZ200; 42% decrease). Moreover, unmutated as well as mutated *PSMB5* expression decreased after exposure to IFN-γ. Specifically, unmutated *PSMB5* expression decreased 23% in 8226/BTZ100 cells and 48% in THP1/BTZ200 cells. Mutated *PSMB5* expression decreased 14% in 8226/BTZ100 and 30% in THP1/BTZ200 cells. Accordingly, both mutated and unmutated *PSMB5* expression declined after IFN-γ exposure, with dominance for unmutated PSMB5 (Additional file 3).

Collectively, bortezomib-resistant hematologic tumor cells possess the capacity to markedly induce immunoproteasome levels upon IFN-γ stimulation, thereby outweighing mutated and unmutated constitutive proteasome levels.

### IFN-γ stimulation confers increased proteasome catalytic activity and HLA Class-I molecule expression

**Figure 3:**
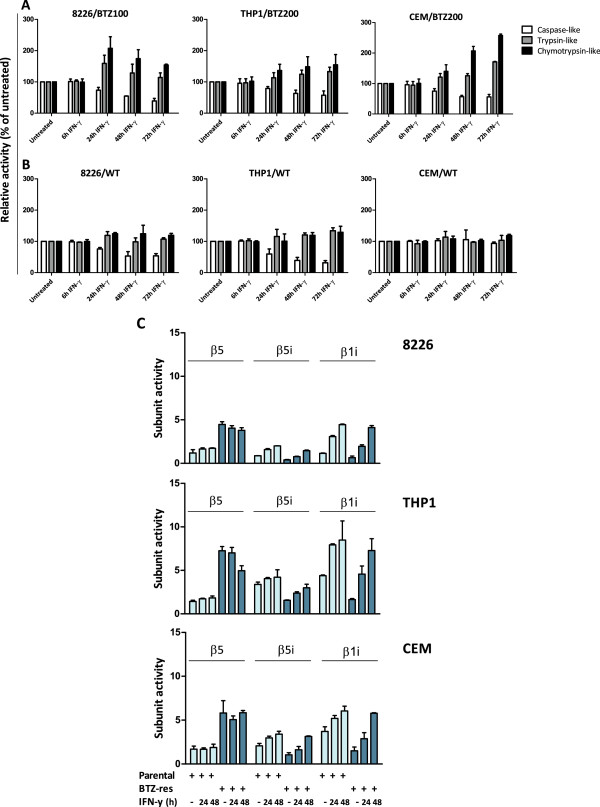
**Impact of IFN-γ exposure on proteasome catalytic activity in intact cells and cell extracts of bortezomib-resistant and bortezomib-sensitive 8226 (MM), THP1 (AML) and CEM (ALL) cells. (A)** Chymotrypsin-like, caspase-like and trypsin-like proteasome activity in intact bortezomib-resistant and **(B)** bortezomib-sensitive 8226, THP1 and CEM cells before and after 6-72 hrs exposure 100 U/ml IFN-γ. Results are presented relative to untreated controls and represent the mean (± SD) of 3 individual experiments. **(C)** β5, β5i and β1i-associated catalytic activity in cell extracts of 8226/WT, 8226/BTZ100, THP1/WT, THP1/BTZ, CEM/WT, and CEM/BTZ200 cells after 24 hr and 48 hr exposure to 100 U/ml IFN-γ. Activity assays in cell extracts employed subunit-specific substrates. Results represent the mean (± SD) of 3 experiments.

To determine whether increased immunoproteasome expression also impacts proteasome catalytic activity, an intact cell-based assay was used to measure the three catalytic proteasome activities in the three bortezomib-resistant cell lines before and after 6-72 h incubation with IFN-γ. Following IFN-γ exposure, chymotrypsin-like proteasome activity increased up to 2.5-fold compared to unstimulated controls, and trypsin-like activity increased up to 1.5-fold, whereas caspase-like activity was slightly decreased (Figure 3A). WT cell lines exposed to IFN-γ displayed only modest increments in chymotrypsin-like and trypsin-like proteasomal activities, whereas caspase-like activity decreased by 50-65% in 8226 and THP1 cells, but not in CEM/WT cells (Figure 3B). Subdividing the chymotrypsin-like activity into β5 and β5i catalytic activities with subunit-specific probes showed that β5 activity did not change in cell extracts of 8226/BTZ100 and CEM/BTZ200 cells upon stimulation with IFN-γ, whereas in THP1/BTZ200 cells the β5 activity declined after 48 h of IFN-γ exposure. For all three parental and bortezomib-resistant cell lines, β5i and β1i activities increased upon stimulation with IFN-γ (Figure 3C). Consistent with increased proteasome catalytic activity, increased expression of cell surface HLA Class I was also observed, particularly in 8226/BTZ100 cells (2-fold) and CEM/BTZ200 and THP1/BTZ200 cells (1.5-fold increase). HLA Class I expression in WT cells was increased moderately (up to 50%) (Additional file 4).

### IFN-γ promotes sensitization of bortezomib-resistant cell lines to cell death by proteasome inhibitors

**Figure 4:**
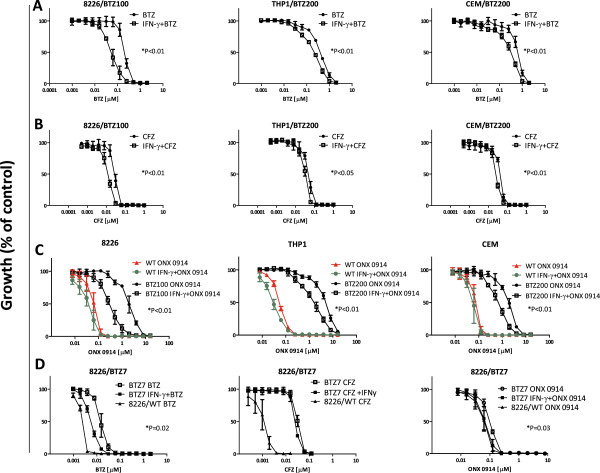
**Sensitivity of bortezomib-resistant cell lines to proteasome inhibitors after IFN-y pre-exposure.** Sensitivity of 8226/BTZ100, THP1/BTZ200 and CEM BTZ/200 cells to **(A)** bortezomib (BTZ) (with and without IFN-γ), **(B)** Carfilzomib (CFZ) (with and without IFN-γ), and **(C)** ONX 0914 (with and without IFN-γ), compared to parental cell sensitivity, and **(D**) and the sensitivity of 8226/BTZ7 cells to BTZ, CFZ, and ONX 0914, as determined by MTT cytotoxicity assays after 4 days drug exposure. Pre-exposure with 100 U/ml IFN-y was for 24h prior to 4-day BTZ, CFZ and ONX 0914 addition. Results represent the mean (± SD) of 3 individual experiments. P-values indicate differences between BTZ-resistant cells exposed or unexposed to IFN-γ.

As we have recently shown that mutated constitutive β5 subunit is a critical factor in conferring resistance to bortezomib [8,9], we hypothesized that IFN-γ-induced upregulation of non-mutated immunoproteasome in bortezomib-resistant cells may re-introduce the targeting capacity of bortezomib and other selective immunoproteasome inhibitors, thereby restoring drug sensitivity. Indeed, cell growth inhibition assays demonstrated that pre-exposure to IFN-γ sensitized 8226/BTZ100 cells 4-fold for bortezomib (Figure 4A), 2-fold for Carfilzomib (CFZ; Figure 4B) and 7-fold for the immunoproteasome inhibitor ONX 0914 (Figure 4C). Similar profiles were observed for THP1/BTZ200 after IFN-γ exposure, though with slightly lower sensitization factors than for 8226/BTZ100 cells (Figure 4A-C). Sensitization factors for bortezomib and carfilzomib were the lowest for CEM/BTZ200 cells, but still 3-fold sensitization for ONX 0914 (Figure 4A-C). IC_50_ values were found to differ significantly between experiments without and with IFN-γ for all high bortezomib-resistant cell lines and drugs. Sensitization impact induced by IFN-γ was further explored in 8226 cells with low levels of bortezomib resistance (8226/BTZ7). Herein, IFN-γ significantly restored parental cell sensitivity to ONX 0914 (p = 0.03) (Figure 4D). For comparison, parental 8226, THP1 and CEM cells were not sensitized or only marginally sensitized to bortezomib, carfilzomib or ONX 0914 after IFN-γ exposure (Additional file 5). Consistently, when PBMCs from healthy individuals were exposed for 24 hours to a concentration range of IFN-γ, they also upregulated immunoproteasome subunits, but did not became sensitized for bortezomib (Additional file 6). A composite summary of the impact of IFN-γ-induced upregulation of immunoproteasomes on the sensitivity of bortezomib-resistant cells to bortezomib, carfilzomib and ONX 0914 is depicted in Additional file 7.

**Figure 5:**
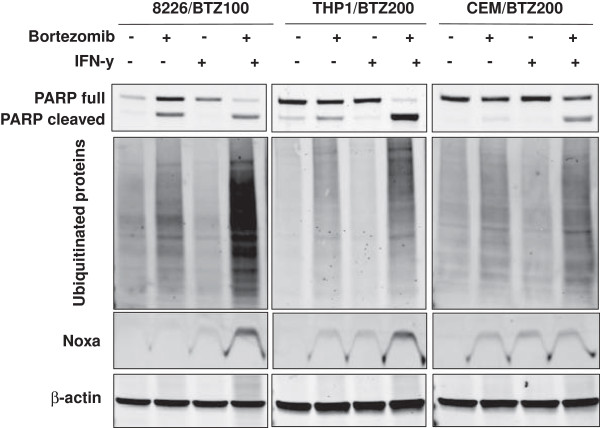
**Apoptosis induction and accumulation of ubiquitinated proteins in bortezomib-resistant 8226 (MM), THP1 (AML) and CEM (ALL) cells after sensitizing cells for bortezomib with IFN-γ.** Western blot analysis of PARP cleavage and expression of NOXA as indicators of apoptosis and accumulation of polyubiquitinated proteins in untreated cells, after 24 h exposure to bortezomib (100 nM for 8226/BTZ100 and 200 nM for both CEM/BTZ200 and THP1/BTZ200), single IFN-γ (100 U/ml), or the combination of IFN-γ and bortezomib.

Restoration of drug sensitivity to (immuno)proteasome inhibitor after IFN-γ exposure was further confirmed by apoptosis induction and activation of PARP cleavage. Western blot analysis revealed a marked increase in cleaved PARP and NOXA expression in bortezomib-resistant cells when bortezomib exposure was preceded by IFN-γ as compared to bortezomib alone (Figure 5). Next, we examined the accumulation of polyubiquitinated proteins as a hallmark of proteasome inhibition in bortezomib-resistant cells upon pre-exposure to IFN-γ followed by incubation with the bortezomib concentration they were stably growing in. Exposure to bortezomib or IFN-γ alone showed a minimal accumulation of polyubiquitinated proteins in bortezomib-resistant cells. In contrast, pre-exposure to IFN-γ in combination with bortezomib introduced a major accumulation of polyubiquitinated proteins pointing to restoration of proteasome inhibitory activity of bortezomib and subsequent induction of apoptosis (Figure 5). Similar results were noted with cells pre-exposed to IFN-γ and subsequently to ONX 0914 for 24 hours (Additional file 8).

### Immunoproteasome subunit β5i is responsible for the sensitization of bortezomib-resistant cell lines

**Figure 6:**
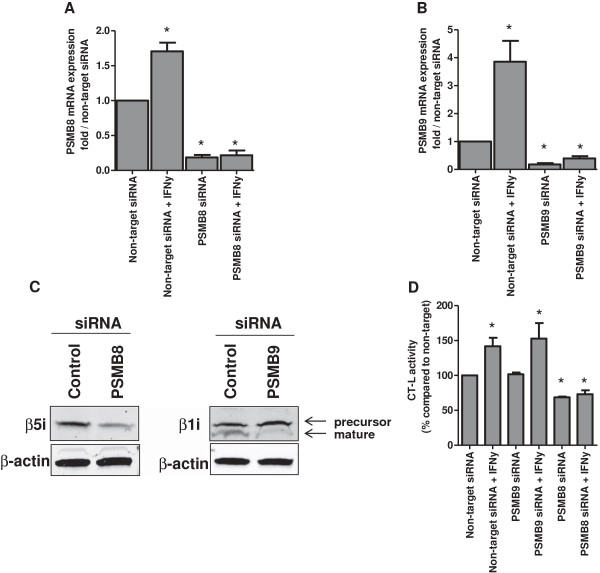
**Effect of IFN-γ after siRNA knockdown of immunosubunits PSMB8 (β5i) and PSMB9 (β1i). (A)***PSMB8* mRNA expression after *PSMB8* siRNA with and without 100 U/ml IFN-γ for 24h compared to non-target siRNA with and without 100 U/ml IFN-y, **(B)***PSMB9* mRNA expression after *PSMB9* siRNA with and without 100 U/ml IFN-γ for 24 h compared to non-target siRNA with and without 100 U/ml IFN-γ. Results are presented as percentage relative to controls (mean (± SD) of 3 individual experiments). **(C)** β5i protein expression after *PSMB8* silencing for 48 h compared to non-target control siRNA (58% downregulation) and protein expression of the mature protein form of β1i after *PSMB9* silencing for 48 h compared to non-target control siRNA (78% downregulation). **(D)** Chymotrypsin-like activity of non-target siRNA as compared to *PSMB8* or *PSMB9* siRNA with and without 100 U/ml IFN-y for 24 h. Results are the means of 4 separate experiments. *P < 0.05

**Figure 7:**
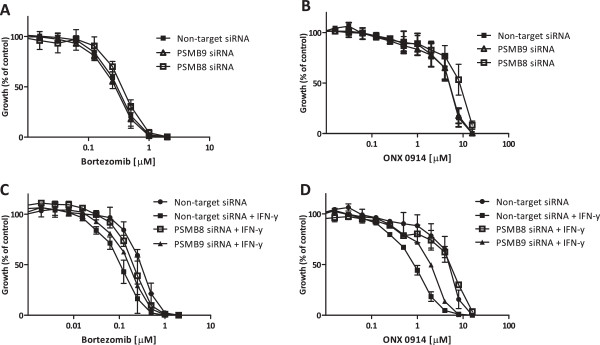
***PSMB8 *(β5i) siRNA attenuates sensitizing effect of IFN-γ to bortezomib and ONX 0914.** Cell growth inhibitory effects of **(A)** bortezomib and **(B)** ONX 0914 after *PSMB8* or *PSMB9* silencing in THP1/BTZ200 cells. Effects of *PSMB8* and *PSMB9* silencing on IFN-y sensitization of growth inhibition of THP1/BTZ200 cells to **(C)** bortezomib and **(D)** ONX 0914. Results depicted are the means (± SD) of 3 independent experiments.

To provide evidence that upregulation of β5i and/or β1i by IFN-γ was responsible for the observed sensitization to proteasome inhibitor, siRNA-dependent downregulation of *PSMB8* (β5i) and *PSMB9* (β1i) was applied in THP1/BTZ200 cells prior to exposure to IFN-γ. Under these conditions, mRNA levels of *PSMB8* and *PSMB9* remained significantly (P = 0.03) suppressed for approximately 80%, even after exposure to IFN-γ, compared to non-target siRNA (Figure 6A-B). Expression of β5i and β1i protein was suppressed for 58% and 78% after 48 h of *PSMB8* and *PSMB9* silencing respectively, compared to non-target control siRNA (Figure 6C). Consistently, after siRNA downregulation of *PSMB8,* but not *PSMB9*, chymotrypsin-like proteasome catalytic activity significantly (P = 0.03) declined to 50% of its control and remained suppressed after exposure to IFN-γ (Figure 6D). Lastly, we determined sensitivity to ONX 0914 and bortezomib with 4-day MTT cytotoxicity assays after *PSMB8* and *PSMB9* silencing with or without IFN-γ pre-exposure. After the sole silencing of *PSMB8*, THP1/BTZ200 cells became slightly more resistant to bortezomib and ONX 0914, while *PSMB9* silencing did not exert any effect (Figure 7A-B). When cells were exposed to IFN-γ after *PSMB8* silencing, bortezomib and ONX 0914 sensitization was attenuated. In contrast, exposure to IFN-γ after silencing of *PSMB9* expression had less effect on bortezomib and ONX 0914 sensitization (Figure 7C-D), indicating that β5i represents the major determinant in exerting apoptosis and growth inhibitory effects of bortezomib and ONX 0914 after exposure to IFN-γ.

## Discussion

The present study is the first to document the impact of IFN-γ on constitutive- and immunoproteasome homeostasis in three bortezomib-resistant tumor cell lines of different hematologic origin and to assess the implications for anti-proliferative activity of proteasome inhibitors. Characteristically, the bortezomib-resistant cell lines largely expressed the mutated form of *PSMB5*, and clearly, IFN-γ increased the expression of catalytically active immunoproteasome levels in bortezomib-resistant cells with concurrent downregulation of both mutated and unmutated alleles of constitutive β5. This property facilitated sensitization to bortezomib, and an even more pronounced sensitization to the immunoproteasome inhibitor ONX 0914. Sensitization effects were most prominent in 8226/BTZ cells and lowest in CEM/BTZ cells, which may be related to the fact that CCRF-CEM leukemia cells have low levels of IFN-γ receptors [30]. At equal doses of IFN-γ, induction of immunoproteasome β5i and β1i subunit mRNA and protein expression was significantly higher (up to 4-fold) in bortezomib-resistant tumor cells compared to parental cells. Concomitantly, constitutive proteasome subunits were clearly downregulated at a protein level, but not as much on mRNA levels. This phenomenon was also reported by others (reviewed by Ebstein et al [31]), indicating that downregulation of constitutive subunits involves a post-transcriptional mechanism. Nonetheless, by employing a very sensitive lightcycler RT-PCR technique, a moderate downregulation on mRNA level was detectable. Additionally, in PBMCs from healthy individuals, the same results were noticed as in the parental cell lines when exposed to IFN-γ, and call for further analyses in bortezomib-resistant patient specimen. It is not clear whether the upregulation of immunoproteasome levels reflects a compensatory and homeostatic effect after initial downregulation during bortezomib resistance development. Importantly, increased β5i expression can drive incorporation of immunoproteasome subunits into prototypic immunoproteasomes [32] or facilitate assembly in hybrid types of proteasomes (β1 + β2 + β5i and β1i + β2 + β5i) [33]. Conceivably, these hybrid forms could compensate for impaired catalytic activity of constitutive proteasomes assembled with a mutated β5-subunit. Following use of β5-selective substrates, chymotrypsin-like catalytic activity in cell extracts of bortezomib-resistant cells increased 2-4 fold over those of parental cells (Figure 3B). These observations are consistent with our previous report in which we observed, using native gel electrophoresis, proficient catalytic capacity of mutated β5 subunit-harboring proteasomes in CEM/BTZ cells for chymotrypsin-like probes, but a diminished inhibitory capacity by bortezomib [9]. Likewise, Kale et al [34] showed that strains of the marine actinobacterium *Salinispora tropica* could maintain self-resistance to the proteasome inhibitor salinosporamide A by expressing a proteasome variant harboring β5 subunit mutations similar to those detected in human THP1/BTZ cells [8,9]. This mutated β5 subunit had retained its capacity to hydrolyze β5-specific substrates, but displayed a diminished sensitivity to inhibition by salinosporamide A.

The expanding knowledge of factors determining bortezomib sensitivity or resistance that emerged from cell line studies (reviewed in ref [35]), still awaits translation and implementation in a clinical setting. With respect to the role of immunoproteasomes, a recent report from our laboratory showed that higher ratios of immunoproteasome over constitutive proteasome in acute leukemia patient samples served as an important parameter for their *ex vivo* sensitivity to bortezomib and ONX 0914 [19]. In addition, Shuqing et al [36] showed an increase in constitutive *PSMB5* mRNA expression in a myeloma patient after bortezomib treatment compared to the pre-treatment sample. Also recently, Leung-Hagensteijn et al [37] showed that immunoproteasome subunit expression was decreased in patients with myeloma tumors resistant to bortezomib, compared to bortezomib-sensitive patients. This study also revealed that the loss of Xbp1 signaling (which is required for plasma cell differentiation and regulation of unfolded protein response) induced bortezomib-resistance in MM cell lines and patient cells. Based on these considerations, strategies that may increase immunoproteasome levels may merit further exploration for therapeutic intervention.

Despite the fact that IFN-γ-induced upregulation of immunoproteasomes facilitates sensitization of bortezomib-resistant cells to bortezomib and ONX 0914, IFN-γ exposure does not establish full restoration of parental sensitivity to bortezomib. This may be due to two reasons; first, inhibition of the catalytic activity of the immunoproteasome alone appears insufficient to exert a cell growth inhibitory effect. Rather, this requires inhibition of chymotrypsin-like activity and co-inhibition of caspase-like or trypsin-like activities [38,39]. Second, the constitutive β5 subunit is structurally altered in all 3 bortezomib-resistant tumor cell lines due to mutations in the *PSMB5* gene introducing single amino acid substitutions (e.g. Ala49Thr) in the bortezomib-binding pocket leading to diminished bortezomib binding efficiency [8,9]. This structural alteration precludes optimal inhibition of the β5 subunit by bortezomib as present in parental cells, thus retaining a significant degree of bortezomib resistance. These considerations specifically apply for cells with a high level (> 100-fold) of bortezomib resistance. In cells (e.g. 8226/BTZ7 cells) with a more clinically relevant low level (~ 5-fold) resistance to bortezomib, IFN-γ exposure reversed 50% of bortezomib resistance and achieved parental sensitivity to ONX 0914 (Figure 4D, Additional file 7). The latter observation is consistent with data from Huber et al [40] who showed that incorporation of immunoproteasome subunits confers structural alterations in the 20S proteasome complex, resulting in improved accessibility of ONX 0914 to the active sites, which would underlie a mechanism for the largest differential sensitizing effect observed with ONX 0914 as compared to bortezomib and carfilzomib.

Knockdown experiments revealed that β5i expression is critically involved in mediating the proteasome inhibitor-sensitizing effects in bortezomib-resistant tumor cells. The role of β5i may first be related to proteasome assembly, in which β5i is required for processing the β1i and β2i subunits [41]. Consistently, β5i deficiency delays immunoproteasome assembly [41]. Beyond increased immunoproteasome subunit expression after IFN-γ exposure, chymotrypsin-like and trypsin-like proteasome catalytic activities were increased, whereas caspase-like activity was decreased. Employing subunit activity-specific probes indicated that the increase in chymotrypsin-like activity was solely accountable for by the increase in β5i but not β5 catalytic activity. At the same time, the decrease in caspase-like activity was solely due to reduced β1 activity since β1i activity actually increased upon stimulation with IFN-γ. Thus, our findings in bortezomib-resistant cells underscore studies showing that replacement of β1 with β1i decreased caspase-like activity and enhanced β5i-associated chymotrypsin-like activity [11,12,42]. Immunologically, a rise in chymotrypsin-like activity would result in the generation of more peptides with hydrophobic C-terminal residues for presentation on MHC class-I molecules [11]. As such, a prominent IFN-γ-induced switch from constitutive to immunoproteasomes in bortezomib-resistant cells could lead to gain of efficiency in antigen presentation by increased peptide loading after immunoproteasome peptide processing. In our study, *PSMB8/*β5i-downregulation resulted in a 50% decrease of chymotrypsin-like activity, whereas β1i downregulation had no effect on any of the three catalytic activities (data not shown). This phenotype seems fully compatible with that of β5i-deficient mice displaying a 50% decrease in the expression of MHC class-I molecules; these alterations were not observed in β1i- or β2i-deficient mice [13,43,44].

Krüger and Kloetzel [45] suggested that IFN-γ induction combines enhanced translational activity with a rapid increase in the pool of polyubiquitinated proteins that require processing by the proteasome. In this context, the IFN-γ-induced synthesis of immunoproteasomes may represent a physiological adaptation to this cytokine-induced oxidative stress. If cells under these conditions were to be exposed to proteasome inhibitors, blocking of functional activity of newly formed immunoproteasomes would result in additional accumulation of polyubiquitinated proteins, causing cell stress and induction of apoptotic cell death. The bortezomib-resistant cell lines did reveal accumulation of polyubiquitinated proteins as in bortezomib-sensitive parental cells but at higher bortezomib concentrations to which they are adjusted [8,9]. This is likely due to an adaptive mechanism in bortezomib-resistant cells to enhance the β5-associated catalytic capacity to process physiological substrates (Figure 3B). Interference with this process by IFN-γ induced upregulation of immunoproteasomes and blocking their function with specific inhibitors could then trigger accumulation of polyubiquitinated proteins and apoptotic cell death, hence being in line with the mechanism proposed by Kruger and Kloetzel [45].

## Conclusion

Downregulation of β5i subunit expression was identified as being an important determinant of acquisition of bortezomib resistance in cell lines of hematologic malignancies. The pharmacological implication of this novel finding is exemplified by the fact that induction of β5i proteasomal assembly after IFN-γ exposure facilitated restoration of sensitivity of bortezomib-resistant cells towards bortezomib and in particular to immunoproteasome inhibitors.

## Competing interests

CJK and JLA are employees of Onyx Pharmaceuticals, Inc.

## Authors’ contributions

DN, JM and JA performed experiments; JLB designed and validated protocols for proteasome subunit specific activity probes. PV performed the statistical analyses. DN and JA analyzed results and prepared the figures; DN, GJ, and JC designed the research and wrote the paper. YGA, CJK, SZ, and GLK discussed the format and content of the article and contributed to the review and editing of the final manuscript. All authors read and approved the final manuscript.

## Supplementary Material

Additional file 1: Table S1 Proteasome subunit primer design and siRNA sequences. Click here for file

Additional file 2: Figure S1 Effect of IFN-γ exposure on constitutive and immunoproteasome subunit protein levels in bortezomib-resistant and bortezomib-sensitive 8226 (MM), THP1 (AML) and CEM (ALL) cells. Expression levels of β5, β1, β2, β5i, β1i and β2i protein were analyzed by ProCISE assay after 6-72 hrs exposure of 8226/BTZ100, THP1/BTZ200, CEM/BTZ200 cells and their parental counter parts to 100 U/ml IFN-γ. Protein levels are expressed in ng subunit/μg total protein and data represent the mean (± SD) of 3 individual experiments. Click here for file

Additional file 3: Figure S2 Quantification of mutated and unmutated *PSMB5* expression in bortezomib-resistant 8226 (MM) and THP1 (AML) cells after IFN-γ exposure. mRNA expression of total *PSMB5* and unmutated *PSMB5* in; (A) 8226/BTZ100 cells compared to parental 8226 cells, (B) THP1/BTZ200 compared to parental THP1 cells. Expression of total *PSMB5*, unmutated *PSMB5,* and *PSMB5* harboring Ala49Thr mutation in (C); 8226/BTZ100 cells exposed to 100 U/ml IFN-γ for 48 hours, and (D); THP1/BTZ200 cells exposed to 100 U/ml IFN-γ for 48 hours, as determined by lightcycler RT-PCR analysis. Click here for file

Additional file 4: Figure S3 Impact of IFN-γ exposure on HLA-Class I expression in bortezomib-resistant and bortezomib-sensitive 8226 (MM), THP1 (AML) and CEM (ALL) cells. HLA-ABC expression after 6-72h IFN-γ exposure in bortezomib-resistant cell lines 8226/BTZ100, CEM/BTZ200, and THP1/BTZ200 and their parental bortezomib-sensitive counterparts. Results represent mean fluorescence index relative to unexposed control cells. Results depict the mean (± SD) of 3 individual experiments. Click here for file

Additional file 5: Figure S4 Sensitivity of parental bortezomib-sensitive cell lines to proteasome inhibitors after IFN-γ pre-exposure. Sensitivity of 8226/WT, THP1/WT and CEM/WT cells to (A) bortezomib (BTZ) (with and without IFN-γ), (B) Carfilzomib (CFZ) (with and without IFN-γ), and (C) ONX 0914 (with and without IFN-γ) as determined by MTT cytotoxicity assays after 4 days drug exposure. Pre-exposure with 100 U/ml IFN-y was for 24 h prior to 4-day bortezomib, carfilzomib and ONX 0914 addition. Results represent the mean (± SD) of 3 individual experiments. Click here for file

Additional file 6: Figure S5 Sensitivity of PBMCs of healthy individuals to bortezomib after IFN-γ pre-exposure and upregulation of immunoproteasome subunits. (A) Sensitivity of PBMCs to bortezomib (with and without 100 U/ml IFN-γ), as determined by MTT cytotoxicity assays after 48 hours of drug exposure. (B) mRNA expression of immunoproteasome subunits *PSMB8* and *PSMB9* upon exposure to various concentrations of IFN-γ for 24 hours. Results represent the mean (± SD) of 3 healthy individuals. Click here for file

Additional file 7: Table S2 Summary effect of IFN-γ exposure on growth inhibition of bortezomib-resistant and bortezomib-sensitive 8226, THP1 and CEM cells by bortezomib, carfilzomib and ONX 0914. Click here for file

Additional file 8: Figure S6 Accumulation of ubiquitinated proteins in bortezomib-resistant 8226 (MM), THP1 (AML) and CEM (ALL) cells after sensitizing cells for ONX 0914 with IFN-γ. Western blot analysis of accumulation of polyubiquitinated proteins in untreated cells, after 24 h exposure to ONX 0914 (250 nM for 8226/BTZ100, 566 nM for CEM/BTZ200 and 1376 nM for THP1/BTZ200), single IFN-γ (100 U/ml), or the combination of IFN-γ and ONX 0914. Click here for file
