# Supplementary figures and images for: Interferon-γ-induced upregulation of immunoproteasome subunit assembly overcomes bortezomib resistance in human hematological cell lines
Source: J Hematol Oncol. 2014 Jan 13;7:7. doi: 10.1186/1756-8722-7-7 (PMC3896789; doi:10.1186/1756-8722-7-7)

A

8226/WT

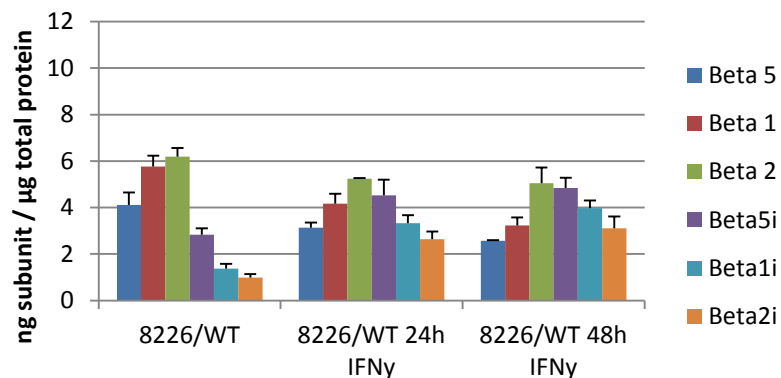

8226/BTZ100

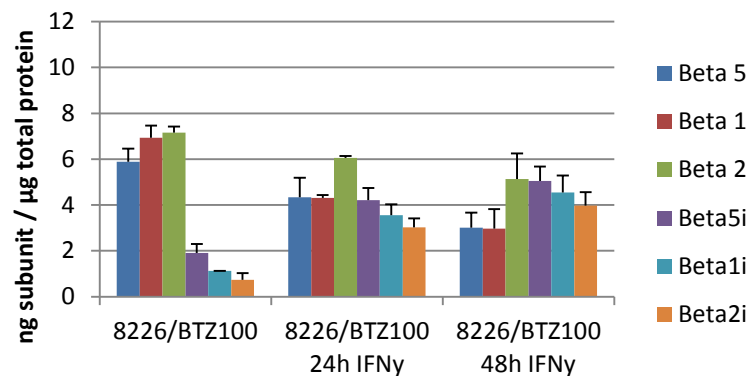

B

THP1/WT

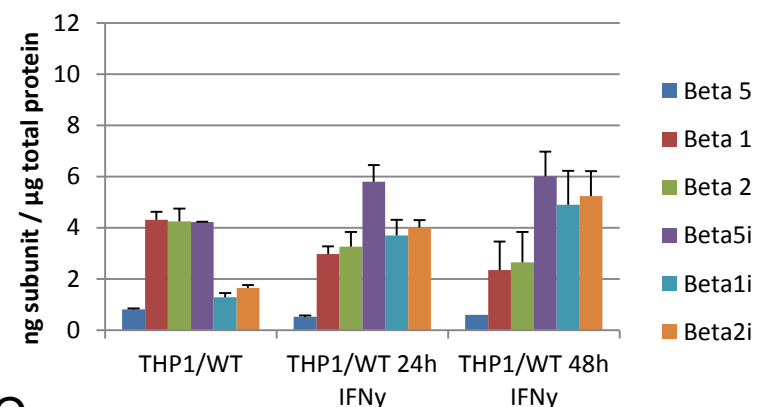

THP1/BTZ200

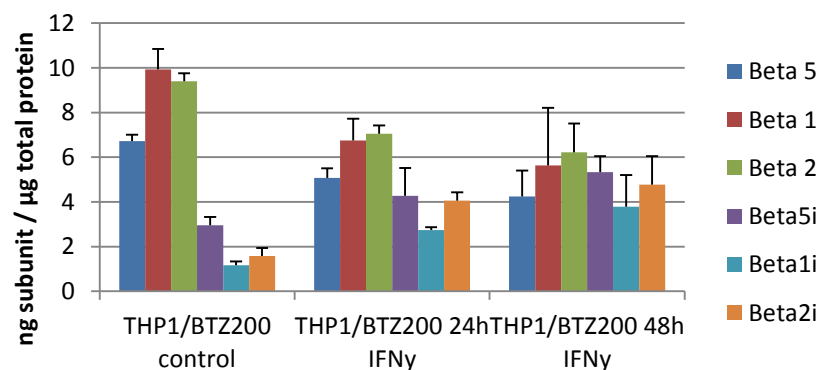

C

CEM/WT

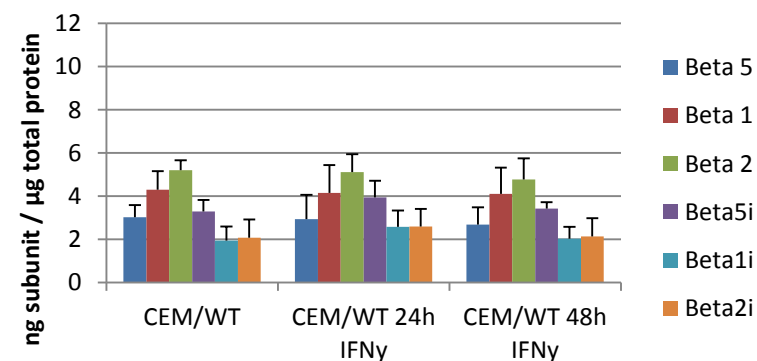

CEM/BTZ200

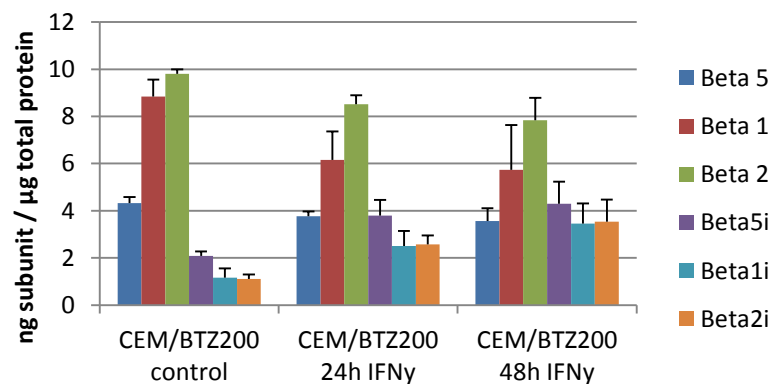

Supplement: Additional file 2: Figure S1 — Effect of IFN-γ exposure on constitutive and immunoproteasome subunit protein levels in bortezomib-resistant and bortezomib-sensitive 8226 (MM), THP1 (AML) and CEM (ALL) cells. Expression levels of β5, β1, β2, β5i, β1i and β2i protein were analyzed by ProCISE assay after 6-72 hrs exposure of 8226/BTZ100, THP1/BTZ200, CEM/BTZ200 cells and their parental counter parts to 100 U/ml IFN-γ. Protein levels are expressed in ng subunit/μg total protein and data represent the mean (± SD) of 3 individual experiments. [file 1756-8722-7-7-S2.pdf]

Figure S2

A

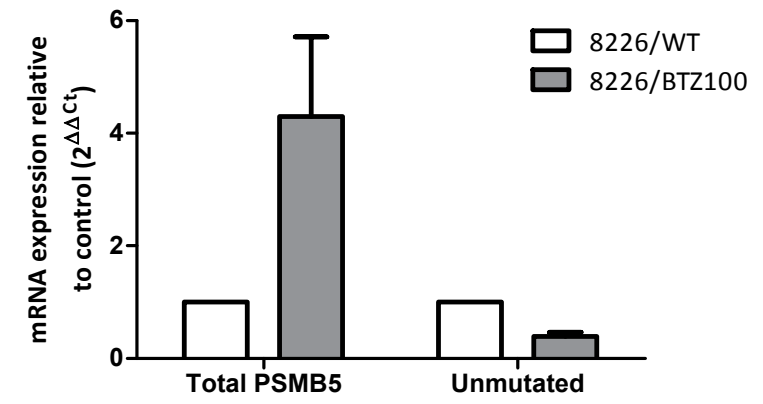

B

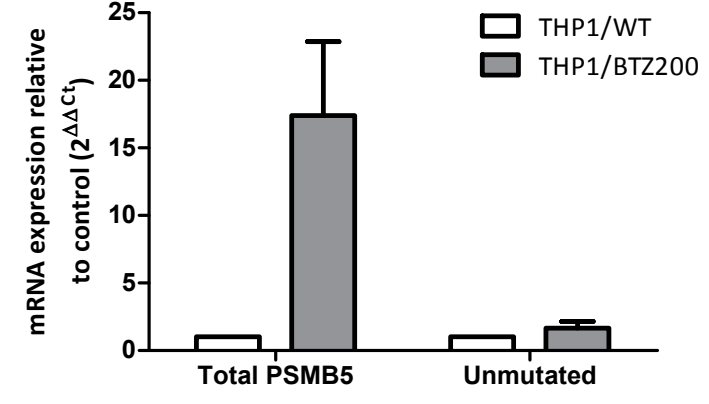

C

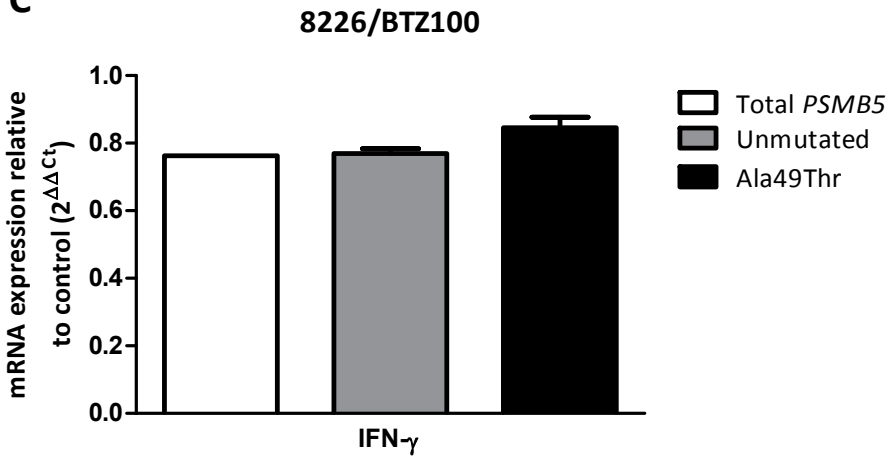

D

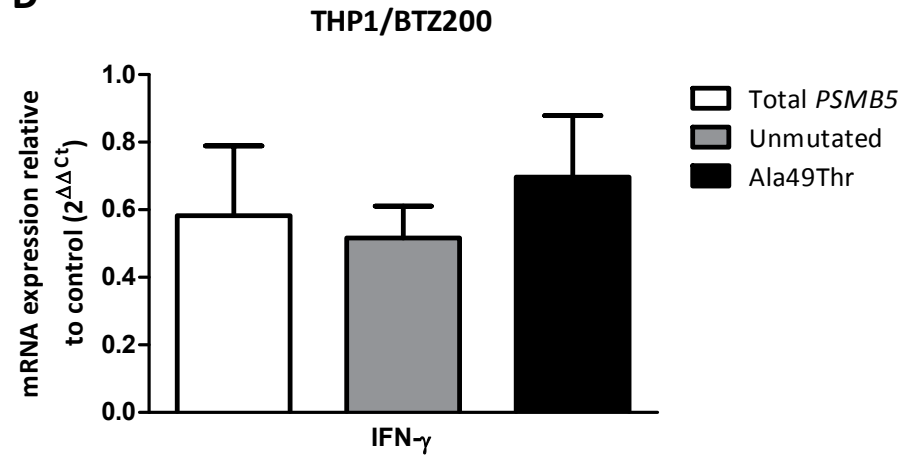

Supplement: Additional file 3: Figure S2 — Quantification of mutated and unmutated PSMB5 expression in bortezomib-resistant 8226 (MM) and THP1 (AML) cells after IFN-γ exposure. mRNA expression of total PSMB5 and unmutated PSMB5 in; (A) 8226/BTZ100 cells compared to parental 8226 cells, (B) THP1/BTZ200 compared to parental THP1 cells. Expression of total PSMB5, unmutated PSMB5, and PSMB5 harboring Ala49Thr mutation in (C); 8226/BTZ100 cells exposed to 100 U/ml IFN-γ for 48 hours, and (D); THP1/BTZ200 cells exposed to 100 U/ml IFN-γ for 48 hours, as determined by lightcycler RT-PCR analysis. [file 1756-8722-7-7-S3.pdf]

Figure S3

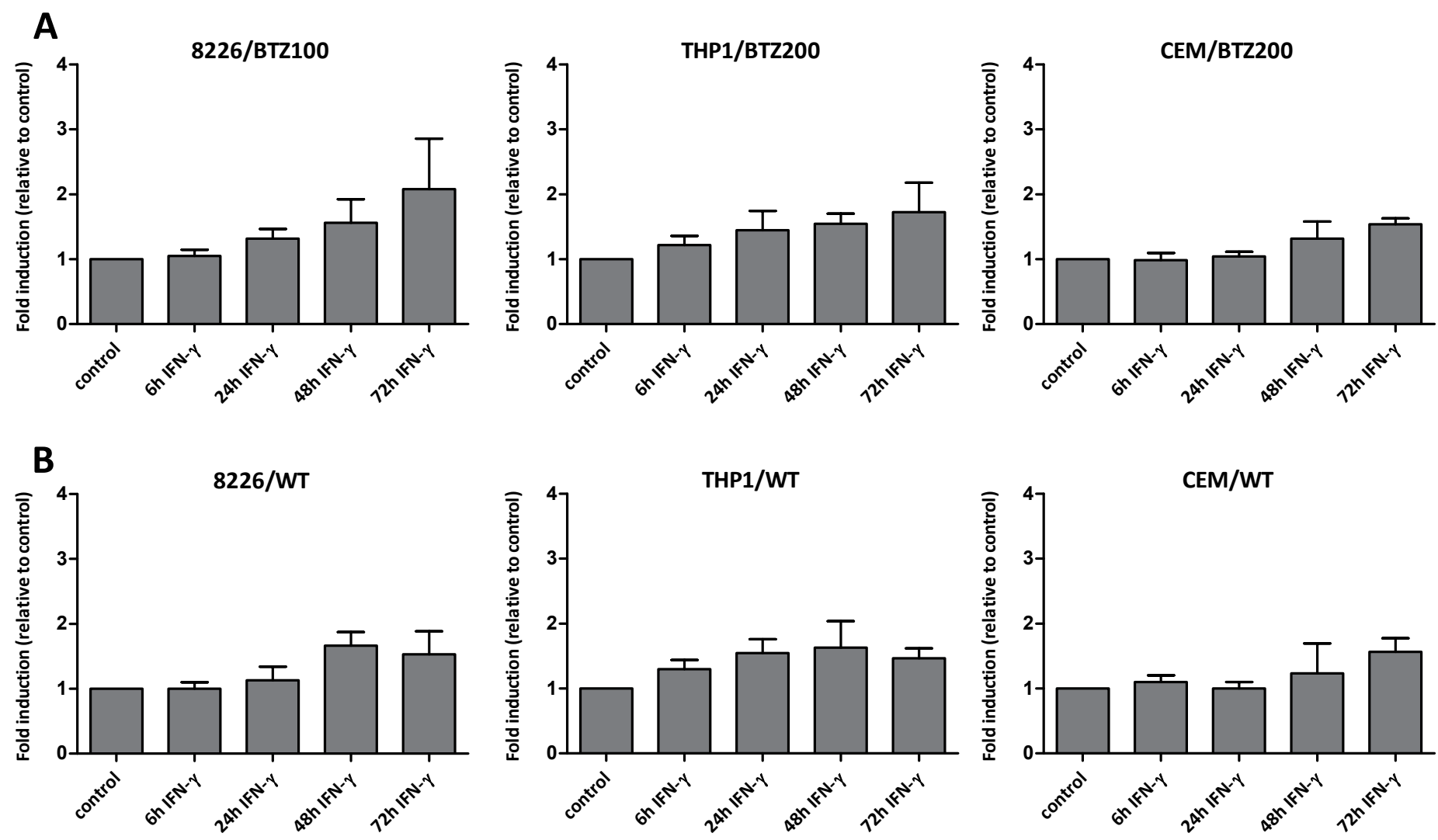

Supplement: Additional file 4: Figure S3 — Impact of IFN-γ exposure on HLA-Class I expression in bortezomib-resistant and bortezomib-sensitive 8226 (MM), THP1 (AML) and CEM (ALL) cells. HLA-ABC expression after 6-72h IFN-γ exposure in bortezomib-resistant cell lines 8226/BTZ100, CEM/BTZ200, and THP1/BTZ200 and their parental bortezomib-sensitive counterparts. Results represent mean fluorescence index relative to unexposed control cells. Results depict the mean (± SD) of 3 individual experiments. [file 1756-8722-7-7-S4.pdf]

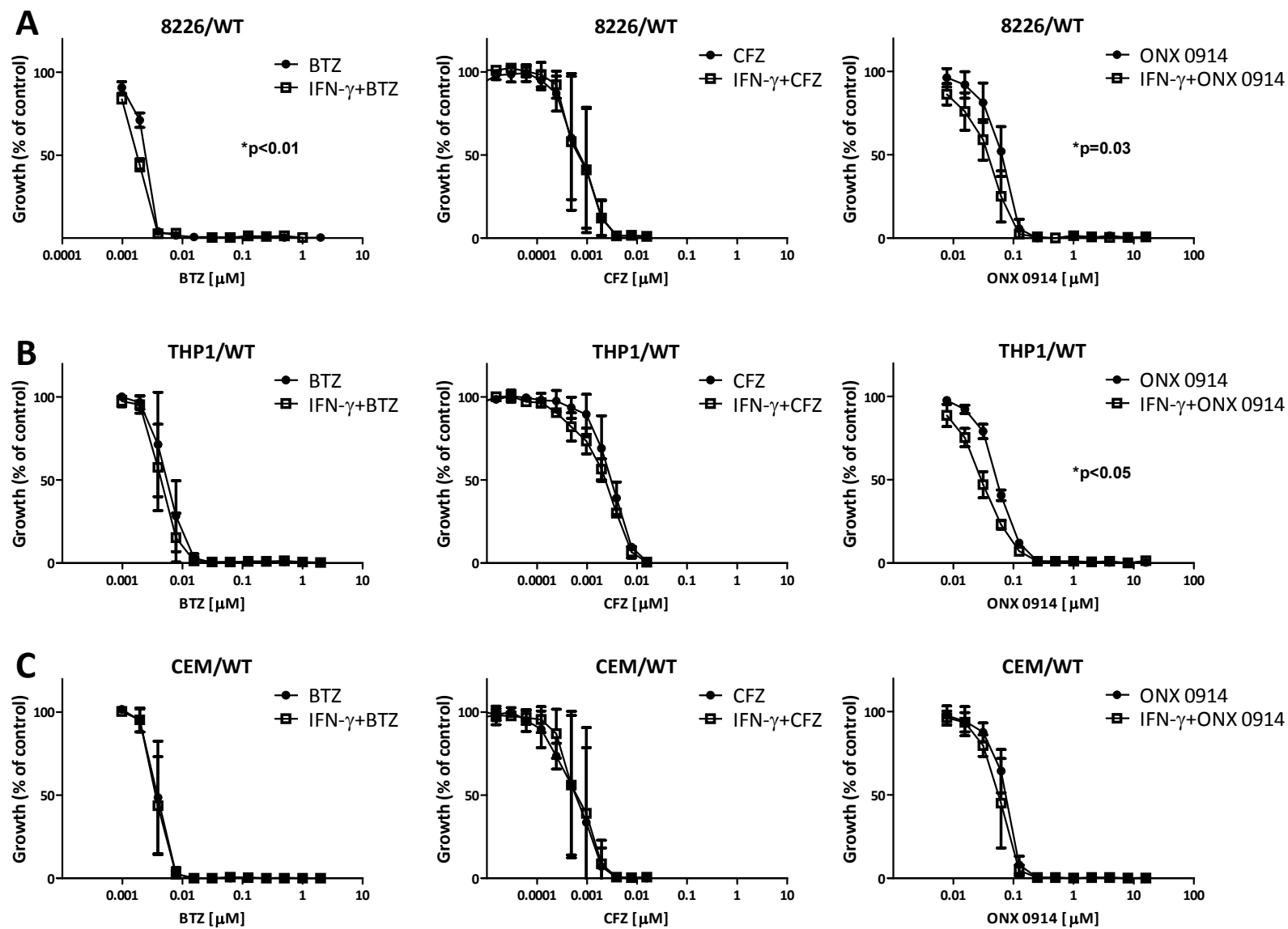

Figure S4

Supplement: Additional file 5: Figure S4 — Sensitivity of parental bortezomib-sensitive cell lines to proteasome inhibitors after IFN-γ pre-exposure. Sensitivity of 8226/WT, THP1/WT and CEM/WT cells to (A) bortezomib (BTZ) (with and without IFN-γ), (B) Carfilzomib (CFZ) (with and without IFN-γ), and (C) ONX 0914 (with and without IFN-γ) as determined by MTT cytotoxicity assays after 4 days drug exposure. Pre-exposure with 100 U/ml IFN-y was for 24 h prior to 4-day bortezomib, carfilzomib and ONX 0914 addition. Results represent the mean (± SD) of 3 individual experiments. [file 1756-8722-7-7-S5.pdf]

Figure S5

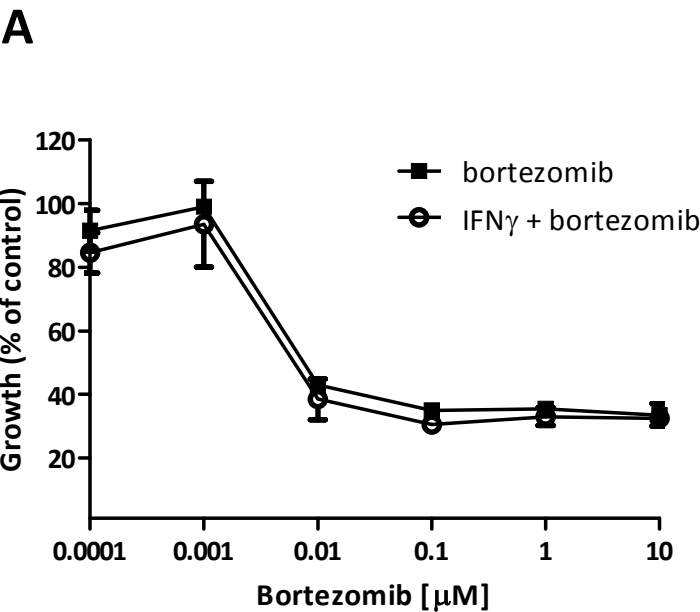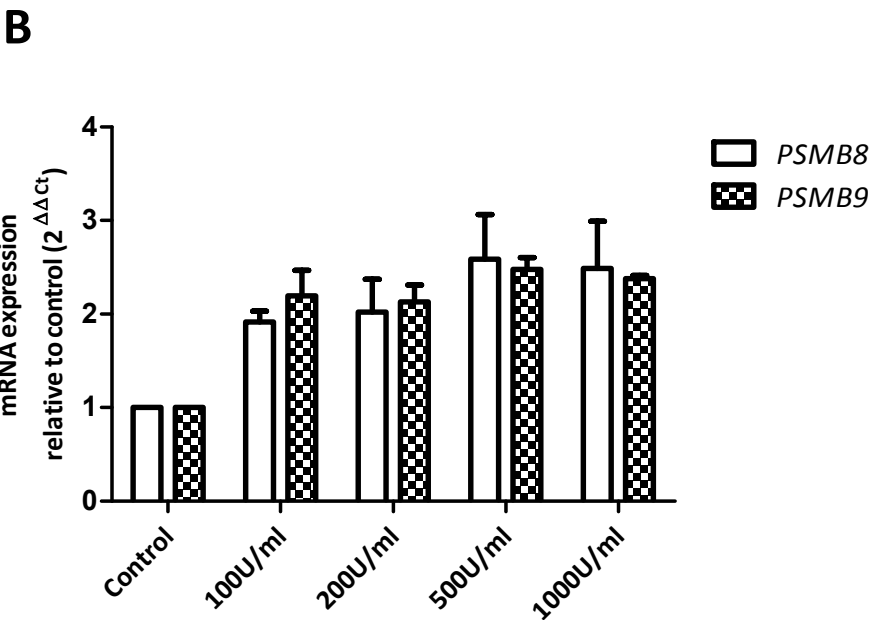

Supplement: Additional file 6: Figure S5 — Sensitivity of PBMCs of healthy individuals to bortezomib after IFN-γ pre-exposure and upregulation of immunoproteasome subunits. (A) Sensitivity of PBMCs to bortezomib (with and without 100 U/ml IFN-γ), as determined by MTT cytotoxicity assays after 48 hours of drug exposure. (B) mRNA expression of immunoproteasome subunits PSMB8 and PSMB9 upon exposure to various concentrations of IFN-γ for 24 hours. Results represent the mean (± SD) of 3 healthy individuals. [file 1756-8722-7-7-S6.pdf]

Figure S6

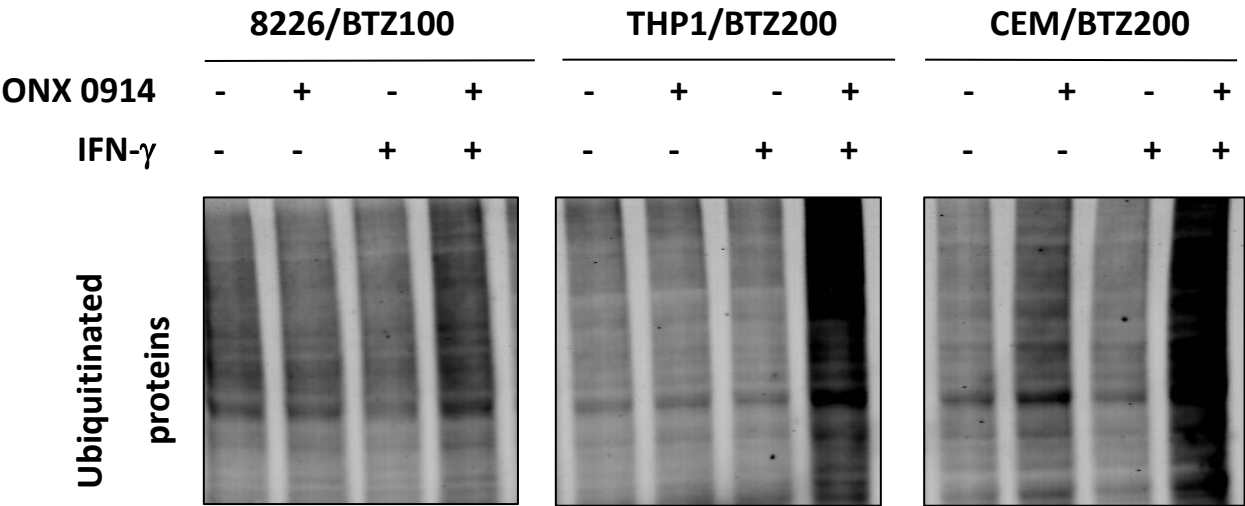

Supplement: Additional file 8: Figure S6 — Accumulation of ubiquitinated proteins in bortezomib-resistant 8226 (MM), THP1 (AML) and CEM (ALL) cells after sensitizing cells for ONX 0914 with IFN-γ. Western blot analysis of accumulation of polyubiquitinated proteins in untreated cells, after 24 h exposure to ONX 0914 (250 nM for 8226/BTZ100, 566 nM for CEM/BTZ200 and 1376 nM for THP1/BTZ200), single IFN-γ (100 U/ml), or the combination of IFN-γ and ONX 0914. [file 1756-8722-7-7-S8.pdf]
